# Seroprevalence of diphtheria toxoid IgG antibodies in the Malaysian population

**DOI:** 10.1186/s12879-021-06285-3

**Published:** 2021-06-16

**Authors:** Ahmad Faudzi Yusoff, Zatil Zahidah Mohd Sharani, Chee Cheong Kee, Nuur Hafizah Md Iderus, Ahmed Syahmi Syafiq Md Zamri, Tharmarajah Nagalingam, Mohd Safrin Mohamad Bashaabidin, Wan Abdul Hannan Wan Ibadullah, Sumarni Mohd Ghazali, Ainur Yusniza Yusof, Yee Ming Ching, Nurhanani Mohamed Nor, Balqis Kamarudin, Norazah Ahmad, Masita Arip

**Affiliations:** 1 SEAMEO TROPMED Malaysia, Institute for Medical Research, National Institutes of Health, Ministry of Health Malaysia, Setia Alam, 40170 Shah Alam, Selangor Malaysia; 2 Biomedical Epidemiology Unit, Institute for Medical Research, National Institutes of Health, Ministry of Health Malaysia, Setia Alam, 40170 Shah Alam, Selangor Malaysia; 3 Sector for Biostatistics and Data Repository, National Institutes of Health, Ministry of Health Malaysia, Setia Alam, 40170 Shah Alam, Selangor Malaysia; 4 Infection Control Unit, Kuala Lumpur Hospital, Ministry of Health Malaysia, Jalan Pahang, 50588 Kuala Lumpur, Malaysia; 5 Allergy and Immunology Research Centre, Institute for Medical Research, National Institutes of Health, Ministry of Health Malaysia, Setia Alam, 40170 Shah Alam, Selangor Malaysia; 6 Infectious Disease Research Centre, Institute for Medical Research, National Institutes of Health, Ministry of Health Malaysia, Setia Alam, 40170 Shah Alam, Selangor Malaysia

**Keywords:** Seroprevalence, Immunization, Diphtheria, Toxoid IgG antibodies, ELISA

## Abstract

**Background:**

Despite high childhood immunization coverage, sporadic cases of diphtheria have been reported in Malaysia in recent years. This study aims to evaluate the seroprevalence of diphtheria among the Malaysian population.

**Methods:**

A total of 3317 respondents age 2 years old to 60 years old were recruited in this study from August to November 2017. Enzyme-linked immunosorbent assay (ELISA) was used to measure the level of IgG antibody against the toxoid of C. diphtheriae in the blood samples of respondents. We classified respondent antibody levels based on WHO definition, as protective (≥0.1 IU/mL) and susceptible (< 0.1 IU/mL) to C. diphtheriae infection.

**Results:**

Among the 3317 respondents, 57% were susceptible (38.1% of children and 65.4% of adults) and 43% (61.9% of children and 34.6% of adults) had protective antibody levels against diphtheria. The mean antibody level peaked among individuals aged 1–2 years old (0.59 IU/mL) and 6–7 years old (0.64 IU/mL) but generally decreased with age, falling below 0.1 IU/mL at around 4–6 years old and after age 20 years old. There was a significant association between age [Children: χ^2^ = 43.22(df = 2),*p* < 0.001)], gender [Adults: χ^2^ = 5.58(df = 1),*p* = 0.018] and ethnicity [Adults: χ^2^ = 21.49(df = 5),*p* = 0.001] with diphtheria toxoid IgG antibody level.

**Conclusions:**

About 57% of the Malaysian population have inadequate immunity against diphtheria infection. This is apparently due to waning immunity following childhood vaccination without repeated booster vaccination in adults. Children at age 5–6 years old are particularly vulnerable to diphtheria infection. The booster vaccination dose normally given at 7 years should be given earlier, and an additional booster dose is recommended for high-risk adults.

## Background

Diphtheria is a vaccine-preventable disease caused by the gram-positive bacteria called *Corynebacterium diphtheriae*. Transmission of the disease occurs by inhalation of respiratory droplets and contact with infected lesions [1] causing respiratory and cutaneous diphtheria respectively. In severe cases, infection may result in systemic diphtheria. The incubation period of respiratory diphtheria normally ranges from 2 to 5 days but can be anywhere between 1 and 10 days. The most common form of diphtheria infection is pharyngeal diphtheria, however other parts of the respiratory tract such as the nasal cavity, the larynx, or a combination of these sites may also be affected. One of the classical presentations of pharyngeal diphtheria is the formation of a pseudo membrane over the nose, tonsils, pharynx and larynx, causing difficulty in breathing and swallowing [2]. Humans are protected against diphtheria infection by the presence of IgG antibodies to diphtheria toxin, induced by vaccination or naturally-acquired after a diphtheria infection [3].

In Malaysia, notification of diphtheria is mandated by the law under the Infectious Disease Prevention and Control Act 1988. The national immunization program was initiated in the early 1950s. The immunization program follows the WHO recommendation where it includes a combination of diphtheria, tetanus, acellular pertussis and inactivated polio vaccines (DTaP), at ages two, three and 5 months, followed by booster doses at 18 months and 7 years respectively. For adults, at present, there is no recommendation for diphtheria booster dose by the Malaysian Ministry of Health. The average childhood immunization coverage rate of above 95% [4] had successfully reduced the incidence of diphtheria from 131 cases in 1980 to below 10 each year in the 1990s [5]. However, in recent years there has been a surge in diphtheria cases. A total of 81 cases of diphtheria were reported from 2016 to 2018 [5] compared to only 10 cases from 2013 to 2015. This could be due to waning diphtheria immunity levels in the population. However, this information is not available as no study has been carried out on this matter. Thus, this study was conducted to determine the sero-prevalence of diphtheria toxoid IgG antibodies in the Malaysian population.

## Methods

### Study design and sampling

This was a cross-sectional study based in government tertiary hospitals. Our study population were individuals or patients attending selected tertiary hospitals in fourteen states in Malaysia during the study period from August to November 2017. The sample size in each state was distributed proportionate to the state’s population size. Two tertiary hospitals were selected in each state with populations over 1.7 million, whereas only one tertiary hospital was chosen for the states with less than 1.7 million population. For states with two selected hospitals, the sample size was equally distributed between the two hospitals. Respondents were recruited from the outpatient, paediatric, medical, obstetrics and gynaecology, and orthopaedic clinics, day care and the blood bank. The age distribution of the respondents was proportionate to the national age distribution. Respondents’ eligibility was limited to Malaysians between 2 to 60 years old. However, those who had fever, were severely ill, had psychiatric illness or were immuno-compromised (HIV, cancer, severe heart disease, liver disease, kidney disease, brain injury and on immunosuppressive drugs) were excluded. If the respondent agreed to participate in the study but refused to either answer the questionnaire or to blood taking, or blood-taking failed after three attempts, they were excluded from the study. The study was performed in accordance with the principles of the Declaration of Helsinki and approved by the Medical Research Ethnics Committee of Ministry of Health, Malaysia.

### Sample size

To our knowledge, there have been no previous studies on the seroprevalence of diphtheria in Malaysia. In 2009, a study was done in the United Kingdom, which found 75% of the population had at least basic protection against diphtheria (≥0.01 IU/ml), compared to 60% in 1996 [6]. Therefore, the required sample size calculated based on estimated 60% seroprevalence of diphtheria in the population, 3% precision and 80% response rate, was 3414.

Written informed consent was obtained from adult respondents, and for children below 16 years old consent was acquired from the parent or guardian. Three ml of venous blood were taken from eligible consented respondents and a brief interviewer-administered questionnaire was used to collect data on age, gender and ethnicity.

### Serological investigation

Blood samples were collected in plain tubes and centrifuged in the respective hospital laboratory. The serum was transferred into Eppendorf tubes and stored in a freezer at − 21 °C prior to transportation to the Institute for Medical Research, Kuala Lumpur for analysis. A commercially available enzyme-linked immunosorbent assay (ELISA) kit (Euroimmun, Lübeck, Germany) was used to measure the level of IgG antibody against the toxoid of *C. diphtheriae* in the blood samples of respondents. Anti-diphtheria toxoid IgG levels were classified using two cut-off points: ≥0.1 IU/mL/< 0.1 (protective/susceptible) [7] and ≥ 0.01/< 0.01 IU/mL (basic protection/no protection). For the latter cut-off point, antibody levels ≥0.01 IU/mL was further categorised as: no protection (< 0.01 IU/mL), uncertain protection (0.01–0.099 IU/mL), immunization protection present (0.1–0.99 IU/mL) and long-term immunization present (≥1.0 IU/mL) [8].

### Statistical analysis

Data analysis was performed using Statistical Package for Social Sciences (IBM SPSS) software. Initially, all the data from the questionnaire and lab result were entered, checked for data entry errors, explored and cleaned. Descriptive analysis was used to describe socio-demographic characteristics of respondents and seroprevalence of anti *C. diphtheriae* toxoid antibody by age (in years). We also calculated the geometric mean diphtheria toxoid antibody level by age. Pearson’s chi square analysis was performed to determine sociodemographic factors associated with diphtheria protection.

## Results

A total of 3493 respondents were recruited in the study, but 5% were excluded because they did not meet the inclusion criteria (i.e., immunocompromised), incomplete interview, refused blood-taking or blood-taking failed after three attempts. The final sample size was 3317 giving a response rate of 95.0% (3317/3493). The demographic composition of the respondents was 41% male, 66.8% Malay, and 32.2% children aged 2–18 years old (Table 1).

**Table 1:** Sociodemographic characteristics of respondents (*n* = 3317)

Variable	n	%
Gender		
Female	1953	58.9
Male	1364	41.1
Ethnicity		
Malay	2213	66.8
Chinese	218	6.6
Indian	339	10.2
Bumiputra Sabah	359	10.8
Bumiputra Sarawak	158	4.8
Peninsular Orang Asli	14	0.4
Others	16	0.5
Age group		
2–6	323	9.7
7–12	366	11.0
13–17	327	9.9
18–30	957	28.9
31–40	581	17.5
41–50	443	13.4
51–60	320	9.6

**Fig. 1:**
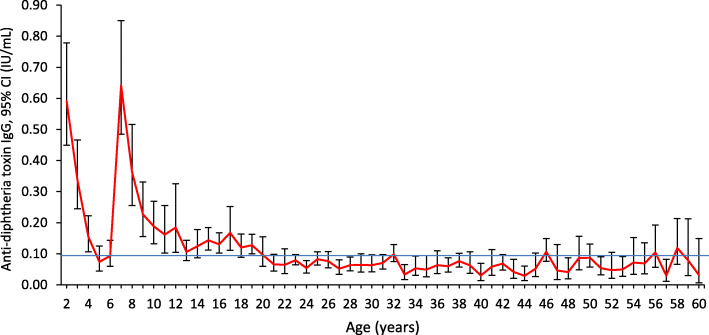
Geometric mean titers and corresponding 95% confidence limits of diphtheria toxoid antibody level by age (2–60 years). (*n* = 3317)

The geometric mean IgG antibody level was high among 2-year-olds (0.59 IU/mL) but decreased to susceptible levels (< 0.1 IU/mL) among 5–6 year olds (0.07 IU/mL). The mean antibody level peaked again at around the age of 7 years old (0.64 IU/mL) and decreased until age 19 years old, thereafter the means were generally below the protective level. Generally, the mean antibody levels decreased with increasing age except at ages 2 and 7 years old (Fig. 1).

Based on WHO classification, overall 57.1% (≥0.1 IU/mL) and 42.9% (< 0.1 IU/mL) of the sampled population were susceptible and protected against diphtheria, respectively. Among the susceptible respondents, 2.4% (0.7% of children and 3.1% of adults) had no protection (< 0.01 IU/mL) against diphtheria and 54.7% (38.1% of children and 62.6% in adults) had uncertain protection (0.01–0.099 IU/mL). While among the protected, 37.6% (49.3 of children and 32.1% of adults) had immunization protection present (0.1–0.99 IU/mL), and 5.3% (11.9% of children and 2.2% of adults) had long-term immunization protection (> 1.0 IU/mL) (Table 2).

**Table 2:** Seroprevalence of diphtheria antibody, overall and by age group

Age group	4-category classification				2-category classification	
	No protection (< 0.01 IU/mL)	Uncertain protection (0.01–0.099 IU/mL)	Immunization protection present (0.1–0.99 IU/mL)	Long-term immunization protection (> = 1.0 IU/mL)	Susceptible (< 0.1 IU/mL)	Protected (> = 0.1 IU/mL)
	n % (95% CI)	n % (95% CI)	n % (95% CI)	n % (95% CI)	n % (95% CI)	n % (95% CI)
Children (2–17 years)	8	379	504	125	387	629
	0.8 (0.4,1.6)	37.3 (34.4,40.3)	49.6 (46.5,52.7)	12.3 (10.4,14.5)	38.1 (35.2,41.1)	61.9 (58.9,64.8)
Adults (18–60 years)	70	1436	744	51	1506	795
	3.0 (2.4,3.8)	62.4 (60.4,64.4)	32.3 (30.5,34.3)	2.2 (1.7,2.9)	65.4 (63.5,67.4)	34.6 (32.6,36.5)
Overall	78	1815	1248	176	1893	1424
	2.4 (1.92.9	54.7 (53.0,56.4)	37.6 (36.0,39.3)	5.3 (4.6,6.1)	57.1 (55.4,58.7)	42.9 (41.3,44.6)

Among the 3317 respondents, 43% (60% of children and 34% of adults) were protected against diphtheria infection. Among adults, adult males had a higher proportion of protection against diphtheria compared to females (*p* = 0.018). By ethnicity, Indians has the highest prevalence of protective antibody level (71.7% children and 45.1% adults protected) among the races. There was a significant association between diphtheria protection and age group among children. Children aged 7–12 years old were most protected (70.2%) followed by age 2–6 (66.3%) and 13–18 (48.2%). In adults, the proportion of protection in the different age groups did not vary significantly, ranging between 33.2 to 35.3% (Table 3).

**Table 3:** Diphtheria protectivity (≥0.1 IU/mL) rates of subjects by demographic characteristics

	All				Children (2–17 years old)				Adults (18–60 years old)			
Variable	Protected		Susceptible		Protected		Susceptible		Protected		Susceptible	
	n	% (95% CI)	n	% (95% CI)	n	% (95% CI)	n	% (95% CI)	n	% (95% CI)	n	% (95% CI)
Overall	1424	42.9 (41.3, 44.6)	1893	57.1 (55.4, 58.7)	629	61.9 (58.9,64.8)	387	38.1 (35.2, 41.1)	795	34.6 (32.6, 36.5)	1506	65.4 (63.5, 67.4)
**Sociodemographic**												
Gender		**								*		
Male	645	47.3 (44.6,49.9)	719	52.7 (50.1, 55.4)	331	62.5 (58.2, 66.5)	199	37.5 (33.5, 41.8)	314	37.6 (34.4, 41.0)	520	62.4 (59.0, 65.6)
Female	779	39.9 (37.7, 42.1)	1174	60.1 (57.9, 62.3)	298	61.3 (56.9, 65.5)	188	38.7 (34.5, 43.1)	481	32.8 (30.4, 35.2)	986	67.2 (64.8, 69.6)
Race		**								**		
Malay	890	40.2 (38.2, 42.3)	1323	59.8 (57.7, 61.8)	405	595 (55.7, 63.1)	276	40.5 (36.9, 44.3)	485	31.7 (29.4, 34.0)	1047	68.3 (66.0, 70.60)
Chinese	88	40.8 (34.5, 47.5)	129	59.2 (52.5, 65.5)	30	56.6 (43.1, 69.6)	23	43.4 (30.4, 56.9)	59	35.8 (28.8, 43.4)	106	64.2 (56.6, 71.2)
Indian	181	53.4 (48.1, 58.6)	158	46.6 (41.4, 51.9)	72	72.7 (63.10, 80.6)	27	27.3 (19.4, 36.9)	109	45.4 (39.2, 51.8)	131	54.6 (48.2, 60.8)
Bumiputera Sabah	175	48.7 (43.6, 53.9)	184	51.3 (46.1, 56.4)	80	64.5 (56.4, 73.2)	44	35.5 (26.8, 43.6)	95	40.4 (34.3, 46.8)	140	59.6 (53.2, 65.7)
Bumiputera Sarawak	77	48.7 (41.0, 56.5)	81	51.3 (43.5, 59)	36	75 (60.9, 85.2)	12	25.0 (14.8, 39.1)	41	37.3 (28.8, 46.7)	69	62.7 (53.3, 71.2)
Others	12	40.0 (24.3, 58.1)	18	60.0 (41.9, 75.7)	6	54.5 (26.8, 79.7)	5	45.5 (20.3,73.2)	6	31.6 (14.9, 54.9)	13	68.4 (45.1, 85.1)
Age group^a^												
2–6	214	66.3 (60.9, 71.2)	109	33.7 (28.8, 39.1)								
7–12	257	70.2 (65.3, 74.7)	109	29.8 (25.3, 34.7)								
13–17	158	48.3 (42.9, 53.7)	169	51.7 (46.3, 57.1)								
18–30	341	35.6 (32.7, 38.7)	616	64.4 (61.3, 67.3)								
31–40	193	33.2 (29.5, 37.2)	388	66.8 (62.8, 70.5)								
41–50	148	33.4 (29.2, 37.9)	295	66.6 (62.1, 70.8)								
51–60	113	35.3 (30.3, 40.7)	207	64.7 (59.3, 69.7)								

* Pearson’s Chi-Square test *p*-value <0.05

** Pearson’s Chi-Square test *p*-value <0.001

^a^ Extended Mantel-Haenszel chi square for linear trend= 178.1, *p*-value <0.001

**Fig. 2:**
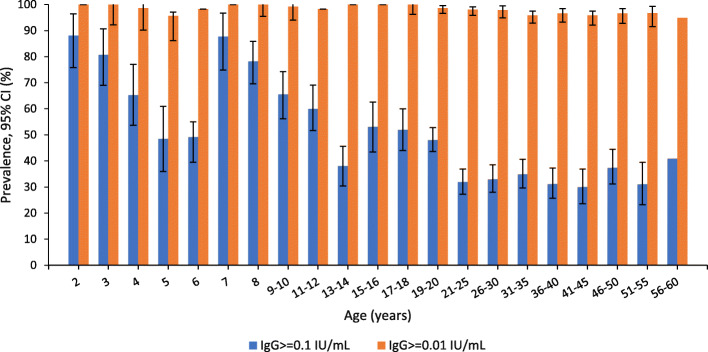
Seroprevalence of protective level of diphtheria antibody by age (2–60 years old) (*n* = 3317)

Using the ≥0.1 IU/mL cut-off point, the proportion of protected was highest among children age 2 years old (88.1%) followed by 7 years old (87.7%). After each peak, the protected proportion dropped drastically as shown in Fig. 2. Protectivity rates among adults ranged from 14.3 to 56.1% (Fig. 2).

## Discussion

Our findings showed that the overall seroprevalence of diphtheria protection was only 43%, in spite of a high immunization coverage rate. The childhood immunization coverage rate in Malaysia in 2017 was 99.3% [4], comparatively better than the global rate of children receiving 3 doses dose of diphtheria primary immunization dose (85%) worldwide in 2019 [9]. This is of great concern as 57% without protection against diphtheria creates an epidemic potential. At the population level, it is believed that vaccine coverage of 80–85% must be maintained in order to maintain herd protection/ community protection and reduce the threat of an outbreak [1, 2]. Whereas in our study, immunity rates among children was only 60% and among adults 34%, both of which were far below the targeted minimum levels. A similar study from Poland in a study population aged 1 month old to 85 years old, showed 61.6% of their population were protected against diphtheria, 63.1% among under 18 year olds and 59.5% among older individuals, with 0.1 IU/ml as the cut-off point for protection [3]. While, in Tajikistan, 51% of their children and young adults were protected against diphtheria [10], 60.5% of Americans 6 years of age or older had fully protective levels of diphtheria antibody (≥0.10 IU/mL) [11] and 66.3% population in China age 3 months old to 74 years old had at least minimal protection (≥0.01 IU/mL) [12]. Our protection level was lower compared to the aforementioned countries. However, it should be noted that vaccination schedules vary between countries, for example in the United States doses are given at 2,4,6 months, 4 years, 19 years and every 10 years subsequently [13]. Whereas in China they are given at 3,4 and 5 months and the last dose at 6 years [13]. Furthermore, variations in the age range of the study population would also result in different prevalences of protection.

Unpublished data from the Malaysian Disease Control Division show very low numbers of diphtheria cases each year, (2016–31 cases, 2017–32 cases, 2018–19 cases). Most of the cases were among children less than 18 years old (70.9, 93.7 and 78.9% of total reported cases respectively). However, our findings show 61.9% of the children below 18 years old were protected. Generally, after three doses of primary diphtheria toxoid immunization, most children achieve antitoxin titers greater than the minimally protective level (< 0.01 IU/ml) [8]. Our study showed 88% of 2-year-olds who should have received 3 primary doses and 1 booster dose as per the immunization schedule, had antibody levels above 0.1 IU/ml. Even though in our study approximately two thirds of were protected, there was a large immunity gap in the 5–6 and 13 years age groups. Less than 50% of children aged 5 to 6 years old had protection against diphtheria and only 35.4% children aged 13 years old were protected. These gaps were also observed in the geometric mean level, where there were dips in the mean at ages 4 to 6 and 13. The high percentage of protectivity against diphtheria, observed at ages 2 and 7 years old was most probably due to the diphtheria booster doses given at the age of 18 months and 7 years old respectively. In the absence of ongoing exposure, immunity wanes over time, requiring booster doses of diphtheria toxoid to maintain diphtheria protective levels.

We found that 34.3% of adult population had full protection (> 0.1 IU/ml) against diphtheria. A study done in Ankara, Turkey among adults age 18 years old and above, reported almost similar seroprevalence of diphtheria of 34.8% [14]. While another study in Turkey reported of 46.3% of adults age 20 to 60 years old had full protection against diphtheria [15]. In China 34.1 to 59.0% of the adult age ≥ 20 years old were protected [12]. Similar results were also seen in a study in Western Europe showed a gradual decrease in the proportion of adults seropositive with age, with the highest proportion of susceptibles in the oldest age groups [16].

Generally, females develop higher immune response to vaccinations [17] however in our study, analysis by gender showed the prevalence of susceptible was significantly higher among female adults. Findings with regards to gender difference in response to diphtheria vaccination have been mixed [18–20]. The possible explanations of sex disparities in immune response are differences in sex hormones and its changes with age, rates of receiving diphtheria vaccination due to occupational related injury and military service, malnutrition and health risk behaviours between males and females [18, 19, 21, 22].

There was a significant association between ethnicity and susceptibility. Genetic variation has been shown to contribute to variability in humoral immunity induced by vaccines [23]. Other studies suggest that ethnic-related genetic factors may play a role in the immune response towards diphtheria vaccination [18, 24]. Differences in age and gender composition as well as health-seeking behaviour between races could also confound this association, however, these variables were not assessed in this study. Therefore, more studies should be conducted to investigate the mechanism underlying this disparity.

There are limitations in our study. Firstly, recruitment of respondents from among patients attending follow-up at the hospitals may result in unequal sampling probability and within hospital population clustering effects were not accounted for. Secondly, we excluded known immunocompromised patients from participating in the study, this may result in underestimation of the proportion of susceptibles. We used ELISA for measuring diphtheria antibody level instead of in vitro toxin neutralization Vero Cell Assay, which is the standard method. Vero cell assay was not performed due to cost and laboratory capacities constraint. Considering the large sample size and time constraint, the ELISA method was employed.

Lastly, respondents’ history of diphtheria vaccination was not verified, hence, the study subjects may include unvaccinated individuals resulting in possible overestimation of the prevalence of susceptibility. Future studies should utilise community-based design for better representation of the general population. This would also allow researchers to access respondents’ health record books for collection of more accurate childhood vaccination history. The strength of this study lies in the large sample size, multi-centre in all states, age-proportionate sampling method and high coverage of diphtheria vaccination in the population.

## Conclusion

A substantial proportion of children under age seven had protective levels, however less than half of children age 5–6 were protected, and antibody levels in adults did not provide sufficient protection against diphtheria. There is a need to re-evaluate the current immunization schedule to prevent diphtheria outbreaks. The diphtheria booster dose should be given earlier at 4–5 years of age instead of at age 7, and a potential booster dose for high-risk adults should be considered.

## Data Availability

The datasets used and analyzed during the current study available from the corresponding author on reasonable request.
